# Surgical indication of iNPH (idiopathic normal pressure hydrocephalus)

**DOI:** 10.1186/2045-8118-12-S1-P25

**Published:** 2015-09-18

**Authors:** Teruo Kimura, Toshihide Sugimura, Shin Fukuda, Makoto Miyano, Masaaki Hashimoto

**Affiliations:** 1 Dohtoh Neurosurgical Hospital, Okhotsk Cerebrovascular Center, Japan

## Introduction

Some patients with iNPH often can recover independent AD and can be easily rehabilitated after shunt surgery. However, other patients sometimes cannot recover independent ADL even if improvement is obtained after treatment, because of some factors. The purpose of this study is to examine the influence of the factor determining the benefit from shunt surgery.

## Methods

All patients underwent a tap test during 9 years from 2002 to 2010, and 154 probable iNPH patients who showed a clinical improvement of at least 10% underwent shunt placement (V-P shunt, 12 patients; L-P shunt, 142 patients). One hundred and thirty-three patients who could be followed up for one year were investigated for age, the interval from onset to surgery, severity, degree of improvement by tap test, MRI findings (DESH or non-DESH).

## Results

According to older patients, the postoperative recovery of mRS, gait disturbance (GD), dysuria, and cognitive impairment (CI) was poor. According to long interval from onset to surgery (group 1<2<3<4<5), the score (mRS, iNPHGS) of them showed higher points mean more severity. There were correlations between the length of the interval from onset to surgery and severity (P<0.01), between the length of the interval from onset to surgery and the degree of improvement after surgery (P<0.01), and between severity and the degree of improvement after shunt (P<0.01). There were correlations between severity of GD (P<0.01) and the degree of improvement after surgery. In tap test, the patient (group A) of remarkable recovery of postoperative score, the symptom was improved, especially, mRS, GD significantly (P<0.01), keeping good ADL after one year (p<0.01). The patient with DESH in preoperative MRI findings (coronal view) was more improvement than patients with non-DESH in mRS, GD, dysuria and CI, significantly (p<0.01), keeping good independent ADL after one year postoperatively.

## Conclusions

Surgical indication is decided with careful consideration of social indication including the comorbidity, fully informed consent should be made.
